# Inhibition of *Candida glabrata* Biofilm by Combined Effect of Dendritic Compounds and Amphotericin

**DOI:** 10.3390/pharmaceutics14081604

**Published:** 2022-07-31

**Authors:** Natalia Gómez-Casanova, Alba Torres-Cano, Alba Xiaohe Elias-Rodriguez, Tania Lozano, Paula Ortega, Rafael Gómez, Jorge Pérez-Serrano, José Luis Copa-Patiño, Irene Heredero-Bermejo

**Affiliations:** 1 Department of Biomedicine and Biotechnology, Faculty of Pharmacy, University of Alcalá, 28871 Alcalá de Henares, Spain; natalia.gomezc@uah.es (N.G.-C.); alba.torresc@edu.uah.es (A.T.-C.); alba.elias@edu.uah.es (A.X.E.-R.); jorge.perez@uah.es (J.P.-S.); josel.copa@uah.es (J.L.C.-P.); 2 Department of Organic and Inorganic Chemistry, Faculty of Pharmacy, Research Institute in Chemistry “Andrés M. del Río” (IQAR), University of Alcalá, 28871 Alcalá de Henares, Spain; tania.lozano@uah.es (T.L.); paula.ortega@uah.es (P.O.); rafael.gomez@uah.es (R.G.); 3 Networking Research Center on Bioengineering, Biomaterials and Nanomedicine (CIBER-BBN), Spain and Institute “Ramón y Cajal” for Health Research (IRYCIS), 28029 Madrid, Spain

**Keywords:** amphotericin, anti-biofilm, *Candida glabrata*, dendritic compounds, confocal microscopy, synergy, treatment

## Abstract

In the last decade, *Candida glabrata* has become an important emerging opportunistic pathogen not only because of the increase in nosocomial infections frequency but also because of its ability to form biofilms and its innate resistance to commercial antifungals. These characteristics make this pathogen a major problem in hospital settings, including problems regarding equipment, and in immunosuppressed patients, who are at high risk for candidemia. Therefore, there is an urgent need for the development of and search for new antifungal drugs. In this study, the efficacy of two dendritic wedges with 4-phenyl butyric acid (PBA) at the focal point and cationic charges on the surface ArCO_2_G_2_(SNMe_3_I)_4_ (1) and ArCO_2_G_3_(SNMe_3_I)_8_ (2) was studied against *C. glabrata* strain to inhibit the formation of biofilms and eliminate established biofilm. For this, MBIC (minimum biofilm inhibitory concentration), MBDC (minimum biofilm damaging concentrations), as well as MFCB (minimum fungicidal concentration in biofilm) and MBEC (minimum biofilm eradicating concentration) were determined. In addition, different combinations of dendrons and amphotericin B were tested to study possible synergistic effects. On the other hand, cytotoxicity studies were performed. *C. glabrata* cells and biofilm structure were visualized by confocal microscopy. ArCO_2_G_2_(SNMe_3_I)_4_ (1) and ArCO_2_G_3_(SNMe_3_I)_8_ (2) dendrons showed both an MBIC of 8 mg/L and a MBDC of 32 mg/L and 64 mg/L, respectively. These dendrons managed to eradicate the entirety of an established biofilm. In combination with the antifungal amphotericin, it was possible to prevent the generation of biofilms and eradicate established biofilms at lower concentrations than those required individually for each compound at these conditions.

## 1. Introduction

*Candida* are one of the main fungal pathogens responsible for invasive infections in humans, especially in immunocompromised patients [1]. The best-known and most frequent species is *Candida albicans*. However, there are other *Candida* non-*albicans* species of clinical relevance that have greater resistance to current antifungal agents, such as *Candida glabrata*, to date the second most isolated species. These facts lead to the failure of therapeutic strategies. This resistance is mainly due to a unique feature of these pathogens, their ability to form biofilms that contributes to their pathogenicity and spread in the healthcare environment, among others [2].

Biofilms are microbial communities adhering to biotic and abiotic surfaces, including medical devices such as heart valves and urinary catheters, and human tissues [1,2,3]. These communities are included in an extracellular matrix that protect cells from different factors such as antifungal agents or host immune system. This matrix acts as a barrier that interferes with and hinders the eradication of infections by *C. glabrata* (or of *C. glabrata* infection), among other species of the genus *Candida*. In these conditions, antifungal effective concentrations are many folds greater than the concentrations required to kill planktonic cells [3]. Furthermore, one of the reasons associated with the high resistance of biofilm related infections and treatment failure is the presence of persister cells, a group of cells within the biofilm that can survive high doses of antifungal agents [4]. 

*Candida* treatment is mainly based on the use of polyenes, such as amphotericin B, or azoles, such as fluconazole. Amphotericin is a broad-spectrum drug used for the systemic treatment of fungal infections; however, it has poor water solubility and limited oral absorption [5]. This compound acts by binding to ergosterol, permeating across the lipid membrane and generating pores. Its activity is fungistatic or fungicidal depending on the concentration of the drug [6]. The problem with these molecules, as with other commercial antifungals, is their cytotoxicity and the tendency to generate resistance that leads to the persistence of *Candida* infections [5]. Consequently, its wide use inevitably causes the emergence of resistant strains that are difficult to treat. Therefore, there is an urgent need to improve the prevention and develop alternative treatment strategies to overcome *Candida* resistance [1]. However, there are difficulties in finding new antimicrobial compounds effective only against *Candida* cells, but not affecting other eukaryotic cells, such as human cells. This fact reduces the number of viable new molecules effective as antifungals.

In this sense, dendritic molecules have emerged as good candidates in the search for new effective antifungals, included against biofilm. Dendrimers are branched nanometric globular polymers with functionalized surface. On the other hand, dendrons start from a focal group that is joined by branches and allow the combination of two therapeutics agents in the same structure (at the focal point and the surface). The use of these structures in the field of biomedicine is widespread. At present, studies have already been reported where the excellent efficacy of these molecules against strains of the *Candida* genus has been confirmed [7,8,9].

Therefore, the present study had the following aims: to determine the activity of cationic dendrons with 4-phenyl butyric acid (PBA); to inhibit the formation of biofilms of *C. glabrata*; to determine the activity needed to eradicate established biofilm; and analyze a combined therapy of dendritic compounds and amphotericin against biofilm formation and established biofilms.

## 2. Materials and Methods

### 2.1. Candida spp. Strains and Growth Conditions

*Candida glabrata* strain 1448 from Colección Española de Cultivos Tipo (CECT 1448) (ATCC 2001) was used in this study. *C. glabrata* was isolated and stored at −80 °C with 20% glycerol (Sigma-Aldrich, Saint Louis, MO, USA) until use. It was grown on Sabouraud chloramphenicol agar (Scharlab, Barcelona, Spain). To stimulate biofilm formation, colonies were transferred into Yeast Extract–Peptone–Dextrose (YPD, 1% Yeast-2% Peptone-2% Dextrose, Scharlab, Barcelona, Spain) and incubated at 37 °C with agitation (150 rpm) for 24 h.

### 2.2. Dendritic Compounds and Amphotericin

**Figure 1:**
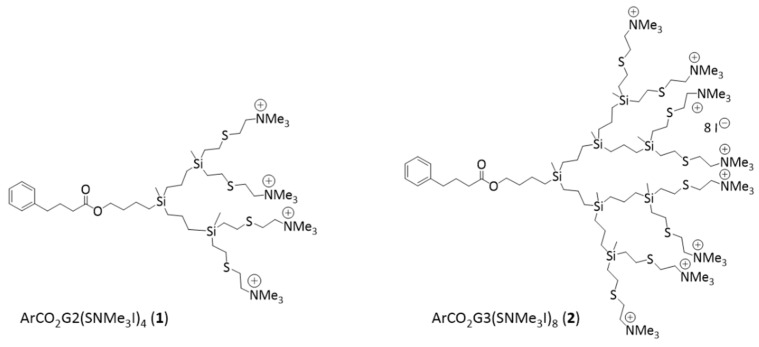
Structures of cationic carbo-silane dendrons ArCO_2_G_2_(SNMe_3_I)_4_ (**1**) and ArCO_2_G_3_(SNMe_3_I)_8_ (**2**).

Two cationic dendritic carbo-silane dendrons (generation 1 and 2), with 4-phenyl butyric acid (PBA) located at the focal point (Figure 1), were tested to study their ability in vitro as anti-biofilm for *C. glabrata.* PBA is a chemical that inhibits endoplasmic reticulum stress and has different applications, such as the treatment of urea cycle disorders [10]. These dendrons are called ArCO_2_G_2_(SNMe_3_I)_4_ (1) (generation 2), and ArCO_2_G_3_(SNMe_3_I)_8_ (2) (generation 3), previously described by [11]. They will be referred as dendron 1 and dendron 2 in the text. All of them were used in previous studies [8]. Two properties were evaluated: the ability to prevent biofilm formation and to eliminate *C. glabrata* established biofilms. Dendrons were tested in 96-well microtiter plates using a series of two-fold dilutions with concentrations ranging from 1 to 512 mg/L. Both compounds were soluble in water. For amphotericin B a stock solution of 250 mg/L was used (Sigma-Aldrich, Saint Louis, MO, USA). The concentrations tested ranged from 0.06 to 128 mg/L. Assays were run in technical triplicates and repeated at least twice in independent experiments. For these experiments, the method of microdilution described in NCCLS document M27-A broth was used [12,13].

### 2.3. Biofilm Inhibition Assay, MBIC and MFCB Determinations

The anti-biofilm activity of dendron 1 and dendron 2 was carried out as previously described [7]. An inoculum of *C. glabrata* was adjusted to 0.5 McFarland standard in RPMI 1640 medium (Sigma-Aldrich) with morpholine-propane-sulfonic acid (Sigma-Aldrich) and 2% glucose (RPMI + MOPS + GLU). Then, 50 μL of the suspension were inoculated in 96-well microtiter plates containing two-fold serial dilutions of the dendrons. Plates were incubated for 48 h at 37 °C. *Candida* controls free of compound, control of compound without inoculum, and control of medium free of compound and inoculum were included. The minimum biofilm inhibitory concentration (MBIC) was determined with resazurin colorimetric assay and has been previously defined as the lowest concentration in which no reduction in resazurin was observed (no growth, nor absorbance signal detected) in biofilm in formation [7,8,14,15]. The minimum fungicidal concentration in biofilm (MFCB) was determined using the drop plate method and was defined as the lowest concentration capable of inducing the total death of the yeast population, avoiding the generation of the biofilm (0% cell viability) [7,8,14,15]. These MFCB values were obtained by scraping the biofilm and plating 5 μL suspension of each well (drop plate method) [7,8].

### 2.4. Biofilm Disruption Assay, MBDC and MBEC Determinations

To determine the ability of the tested dendrons to disrupt established biofilms, the biofilms were formed in microtiter plates as follows. An inoculum of *C. glabrata* was adjusted to 0.5 McFarland standard in RPMI + MOPS + GLU. Then, 100 μL of the suspension was inoculated in 96-well microtiter plates. Plates were incubated for 48 h at 37 °C. Then, the medium was removed to eliminate non-adherent cells and wells were washed with sterile PBS (Phosphate Buffered Saline). Then, 100 µL of serial concentrations of dendron 1 and dendron 2 were added to each well to assess their ability to eradicate established biofilms. Plates were incubated for 48 h at 37 °C. *Candida* controls free of compound, control of compound without inoculum, and control of medium free of compound and inoculum were included. The minimum biofilm damaging concentrations (MBDC) were determined with resazurin colorimetric assay and has previously been defined as the lowest concentration at which no reduction in resazurin was observed (no growth, nor absorbance signal detected) in established biofilm [7,8,14,15]. The minimum biofilm eradicating concentration (MBEC) using the drop plate method was defined as the lowest concentration capable of inducing the total death of the previously established biofilm (0% cell viability) [7,8,14,15,16]. These MBEC value was obtained by scraping the biofilm and plating 5 μL suspension from each well (drop plate method) [7,8].

### 2.5. Combination Therapy of Dendritic Compounds and Amphotericin against C. glabrata Biofilm

The antifungal activity of the combination of cationic carbo-silane dendrons with amphotericin has been studied to evaluate the existence of a cooperative effect between them against *C. glabrata* biofilms. The anti-biofilm activity of the dendrons for inhibition of biofilm formation and elimination of established biofilm was studied. Preventing biofilm formation, a concentration ranging from 0.0035 mg/L to 0.125 mg/L was used for amphotericin and from 0.25 mg/L to 8 mg/L for dendron 1 and dendron 2. To establish the biofilm assay, a concentration ranging from 0.125 mg/L to 4 mg/L was used for amphotericin and from 4 mg/L to 128 mg/L for dendron 1 and for dendron 2. Then, the fractional inhibitory concentration index (FICI) was calculated to evaluate the synergistic activity using the formula:

FICI = ((minimum concentration of drug A in combination)/(minimum concentration of drug A alone)] + [(minimum concentration of drug B in combination)/(minimum concentration of drug B alone)).

Data interpretation: FICI ≤ 0.5 indicates synergy, 0.5 < FICI ≤ 1 additive, 1 < FICI < 4 indifference, FICI ≥ 4 antagonism [17].

### 2.6. Resazurin Assay

Resazurin (Sigma-Aldrich) solution at 0.01% (*w/v*) was filtered and conserved at 4 °C [7,18,19]. After the incubation time, the wells of the treated microplates were washed with PBS. Then, 100 μL of PBS and 20 μL resazurin solution were added to each well. Plates were incubated in the dark at 37 °C for 24 h. Absorbance was measured at 570 and 600 nm in a microplate reader (Epoch^TM^, BioTek, TX, USA) [7,8]. This method allowed obtaining the MBIC values and the MBDC values (in the biofilm formation and in the stabilized biofilm experiment, respectively).

### 2.7. Drop plate Method

Biofilms were scraped and homogenized by shaking with the micropipette several times and 5 μL of well suspensions were transferred onto Chloramphenicol-Sabouraud agar plates [20]. Plates were incubated for 48 h at 37 °C. These values were determined at concentrations where growth was not observed. This method allowed obtaining the MFCB values and the MBEC values (in the biofilm formation and in the stabilized biofilm experiment, respectively).

### 2.8. Determination of the Degree of Cytotoxicity of Dendrons in Combination with Amphotericin

HeLa cells (ATCC^®^ CCL-2^TM^) were used to evaluate the cytotoxicity of dendrons in combination with amphotericin (concentrations that produce synergistic or additive effect). Assays were performed in 24-well plates (NUNC^TM^) in Dulbecco’s Modified Eagle Medium supplemented with fetal bovine serum (10%) and 1% antibiotic mix: 10,000 U penicillin, 10 mg streptomycin and 25 μg amphotericin per mL (Sigma-Aldrich Ltd.). Cells were seeded at a density of 1 × 10^4^ cells/well in 300 μL of fresh medium. For 5 days, plates were incubated at 37 °C in a 5% CO_2_ atmosphere. Then, the medium was replaced by 300 μL of single compounds (dendron 1, dendron 2 and amphotericin) or combinations of these with amphotericin diluted in fresh medium. Control wells received only 300 µL of fresh medium. After 48 h of incubation, wells were washed with PBS (three times), and 500 μL of medium were added to each well. Then, each well received 50 μL (5 mg/mL) of microculture tetrazolium (Sigma-Aldrich Ltd.) and plates were incubated for 4 h at 37 °C. Finally, medium was discarded and 500 μL of dimethyl sulfoxide were added to dissolve formazan crystals. Absorbance was measured at 570–630 nm in a microplate reader (BioTek Instruments Inc. Model: ELX 800). Results: values <10% were non-cytotoxic, values 10–25% were low cytotoxicity, and values 25–40% were moderate cytotoxicity levels [21]. 

### 2.9. Confocal Laser Scanner Microscope

Confocal laser scanning microscopy (CLSM) was used to visualize the damage produced by the compounds under study. The cell suspensions obtained from biofilms of *C. glabrata* treated with dendrons, amphotericin and in combination were visualized by CLSM using 1% propidium iodide (PI) (Merck KGaA, Darmstadt, Germany). The samples were incubated in the dark for 15 min. Dead cells were stained in red. A LEICA TCS-SL or SP5 Confocal Laser Scanning Microscope was used, using argon and helium/neon ion lasers. The excitation/emission range for PI is 490/635 nm.

### 2.10. Data Analysis

All analyses were carried out using the GraphPad Prism 9 program for Windows (GraphPad Software, 2021, San Diego, CA, USA).

## 3. Results and Discussion

### 3.1. Effect of Dendritic Compounds on Biofilm Development and Formation

Due to the importance of the biofilm resistance, different research groups are actively working to find new molecules capable of destroying biofilms. Some of their results show the activity of biodegradable silver nanoparticles [22], silymarin, which is obtained from the shells of *Silybum marianum* seeds [23], or the essential oil from the Sardinian endemic *Juniperus oxycedrus* L. ssp. macrocarpa aerial parts [24]. 

**Figure 2:**
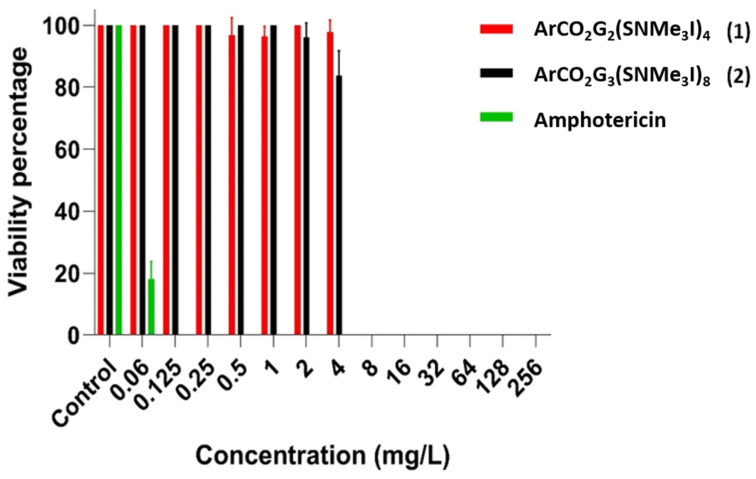
Representation the viability percentage of *C. glabrata* for dendron 1, dendron 2 and amphotericin after the treatment of a biofilm in formation.

In the present study, dendritic systems were used. Within these systems we can differentiate a type of compound, dendrons, dendrimer sections with different terminal groups and a focal point. In this study, dendrons with PBA in the focal point were used. The results obtained with the resazurin colorimetric method showed that dendron 1 and dendron 2 had a MBIC of 8 mg/L, and the amphotericin a MBIC of 0.125 mg/L (Table 1). No gradual decrease was observed in viability as the concentrations tested increased in the case of dendrons and amphotericin (Figure 2). On the other hand, the drop plate method showed that dendron 1 and dendron 2 had a MFCB of between 8 and 16 mg/L, and amphotericin showed a MFCB of 0.125 mg/L (Table 1).

Previous studies have demonstrated the effectiveness of the tested compounds against *C. albicans* CECT 1002 [8]. In these studies, the dendron 1 showed a MBIC and MFCB of 16 mg/L. The new data obtained in the present study support the excellent anti-biofilm activity/effectiveness of this dendron against different species of the genus *Candida*, the MBIC value even lower against *C. glabrata* CETC 1448 (8 mg/L). Related to dendron 2, our results showed that it was effective against *C. glabrata* (MBIC of 8 mg/L and MFCB of 8–16 mg/L). Therefore, this strain was even more sensitive to dendron 2 than *C. albicans* in preventing biofilm formation (MBIC and MFCB of 256 mg/L). Furthermore, the results obtained for the MBIC and MFCB for both dendritic compounds were not different in *C. glabrata*, unlike in *C. albicans,* so that specific characteristics of the cell wall of *C. albicans* could prevent the affinity of this larger molecule (dendron 2) to the cell membrane. 

### 3.2. Effect of Dendritic Compounds on Eradication of Established Biofilms 

**Figure 3:**
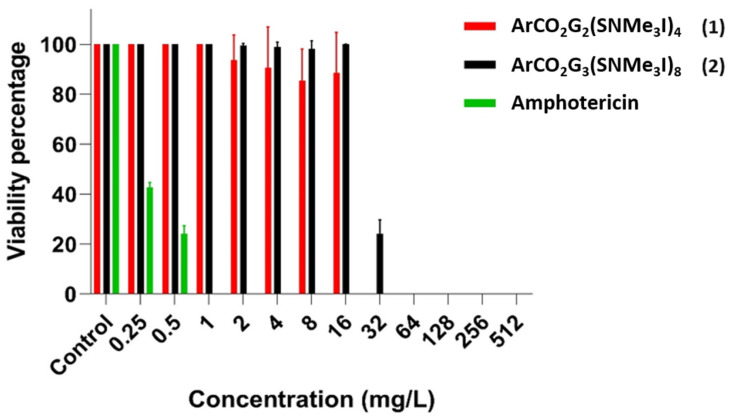
Representation the viability percentage of *C. glabrata* for dendron 1, dendron 2 and amphotericin after the treatment of established biofilms.

Data obtained with the resazurin colorimetric method showed that dendron 1 presented a MBDC of 32 mg/L, dendron 2 a MBDC of 64 mg/L and amphotericin a MBDC of 1 mg/L (Table 1). The non-reduction of resazurin confirmed the low metabolic activity of *C. glabrata* cells forming biofilms at the mentioned MBDC concentrations. Therefore, the viability of a large part of the biofilm cells might be altered after treatment with these concentrations. Amphotericin treatments showed a gradual decrease in viability as concentration increased (Figure 3), and dendron 1 drastically reduced viability from around 100% to 0%. Regarding dendron 2, although the MBDC was 64 mg/L, a significant reduction in the viability of the cells that formed the biofilm was observed at 32 mg/L. On the other hand, the drop plate method showed that dendron 1 had a MBEC of between 256 and 512 mg/L and dendron 2 had a MBEC of 512 mg/L. In the case of amphotericin, its MBEC could not be determined, since colonies of *C. glabrata* grew on the agar plates at all the concentrations tested (Table 1). In addition, although the MBEC concentrations were higher, a substantial reduction was observed in the number of colonies grown in the drop plates experiment compared to the control, from 32–64 mg/L (MBDC) to 512 mg/L (MBEC) for both dendrons, dendron 1 and dendron 2. This fact is interesting for future experiments, in order to try to eliminate these resistant cells using combination therapy with other antifungals and using repeated application over time (new doses every 24 h) with the main objective of completely eliminating biofilms. 

In established biofilms of *C. albicans* CECT 1002, previous studies also demonstrated their activity [8]. Dendron 1 was again much better against *C. glabrata* (MBDC of 32 mg/L) than against *C. albicans* (MBDC of 64 mg/L). In addition, the main difference that we have found in the present study was that both dendrons, dendron 1 and dendron 2, did manage to eradicate the previously established biofilm and no resistant cells were found. However, even though these compounds were effective in damaging the biofilms of *C. albicans*, neither of these molecules managed to eliminate some resistant cells that could be hetero-resistant or persister cells. These types of cells are a recognized problem associated with high resistance of biofilms and the incidence of biofilm-related infections, because this group of cells can survive against high doses of antifungals [4,25]. 

### 3.3. Combination Therapy of Dendritic Compounds and Amphotericin against C. glabrata Biofilm Formation 

The combination of new molecules with commercial antifungal agents is a widely used procedure that improves activity and solves the problem associated with intrinsic resistance among *Candida*. Besides, it is difficult to find effective antifungals at low and non-cytotoxic concentrations to treat *Candida* infections. Therefore, the use of combination therapy allows the reduction of both compounds to obtain the same, or even better, results than if they were used at higher concentrations, for example, the use of tyrosol with amphotericin [26], acetylsalicylic acid with amphotericin [27], tyrocidines with amphotericin or caspofungin [28] and pseudolaric acid B with fluconazole [29]. Combinations of both compounds, dendron 1 or dendron 2, with amphotericin at different concentrations were studied and the FICI was calculated, as well as the viability percentage (Table 2). 

Combination therapy studies carried out against biofilm formation gave satisfactory results. The best results were obtained by dendron 2, but combinations with dendron 1 only showed an additive effect (Table 2). We observed one effective combination that was able to prevent *C. glabrata* biofilm formation. This combination showed a FICI value of 0.5 (synergistic effect): 2 mg/L of the dendron 2 with 0.03 mg/L of amphotericin (FICI = 0.49) (Table 2). It should be noticed that a combination therapy approach managed to reduce the compounds’ effective concentrations against biofilm development, from 2 to 8 times lower than concentrations required in individual treatments. In addition, although other concentrations did not show synergy, they showed an additive effect and managed to reduce the value of their individual MBICs, such as 0.25 mg/L of the dendron 2 with 0.06 mg/L of amphotericin (FICI = 0.51, additive) (shown in Table 2). In addition, the results obtained on agar plates experiments (drop plate method) confirmed the absence of growth at the combination of 4 mg/L of dendron 2 with 0.06 mg/L of amphotericin. Therefore, we were able to improve the antifungal activity of amphotericin, reducing the MBICs concentration for both compounds (dendron 2 and amphotericin), and also their MFCBs. 

On the other hand, the synergistic study with dendron 1 and amphotericin showed that these combinations were not as effective in preventing *C. glabrata* biofilm formation as those combinations with dendron 2. In addition, there were no combinations that resulted in a synergy FICI value. However, although the FICI values were greater than 0.5, zero percent viability was achieved by reducing the concentrations of the individual MBICs (Table 2). The data obtained in the agar plates experiments (drop plate method) confirmed that the most effective combination was 4 mg/L of the dendron 1 with 0.06 mg/L of amphotericin, since it was not only possible to reduce the individual MBICs, but also their MFCBs (Table 2).

The stress generated by the combination treatments induced a growth reduction on agar plates, observed as a reduction in colony number and size. The results were confirmed after 48 h of incubation at optimal growth conditions.

### 3.4. Combination Therapy of Dendritic Compounds and Amphotericin against C. glabrata Established Biofilm 

The complete eradication of the cells that form an established biofilm is difficult to achieve due to the properties of these communities. Commonly, when compounds capable of killing all cells in a biofilm are found, a high dose of the compound is required. This affirmation was confirmed in our study as exposed in Section 3.2. Likewise, we cannot forget that the use of a high concentration of compounds can be harmful not only to the microorganism, but also to human cells. For this reason, conducting synergy studies is essential, not only to reduce effective concentrations but also to reduce the appearance of resistant strains. Our data confirmed that the synergistic activity of both compounds (dendron 1 or dendron 2) with amphotericin could eliminate all viable cells of an established *C. glabrata* biofilm. For dendron 1, we registered a synergistic effect for the combination of 128 mg/L of the dendron 1 (individual MBEC of 256–512 mg/L) with 4 mg/L amphotericin (no individual MBEC value determined), and 128 mg/L the dendron 1 with 1 mg/L amphotericin. The use of these combinations managed to eliminate 100% of viable cells of the established *C. glabrata* biofilm. For dendron 2, we only registered a synergistic effect at the concentration of 128 mg/L of the dendron 2 (individual MBEC of 512 mg/L) with 4 mg/L amphotericin (no individual MBEC value determined), that managed to eradicate the biofilm structure completely. The synergistic effect was especially reflected in the behaviour of amphotericin (no individual MBEC value for the concentrations studied), the compound that was reduced. In the non-active combinations tested (without/non biofilm eradication), it was observed that the stress generated by the combination treatments induced a growth reduction on agar plates, observed as a reduction in colony number and size. The results were confirmed after two days of incubation at optimal growth conditions.

### 3.5. Cytotoxicity 

The cytotoxicity results of the compounds obtained individually have been previously reported (cell viability < 60%) [8]. For this study, the cytotoxicity of the combinations with amphotericin which showed a better activity was assessed on HeLa cells (synergy and additive effect (Table 2)). It is common that the synergistic use of two compounds not only provides good antimicrobial activity, but also a reduction in compound concentrations and in their cytotoxicity. However, in this study, none of the active combinations reduced their cytotoxicity (cell viability < 60% for the combinations tested).

### 3.6. Confocal Microscopy in C. glabrata 

**Figure 4:**
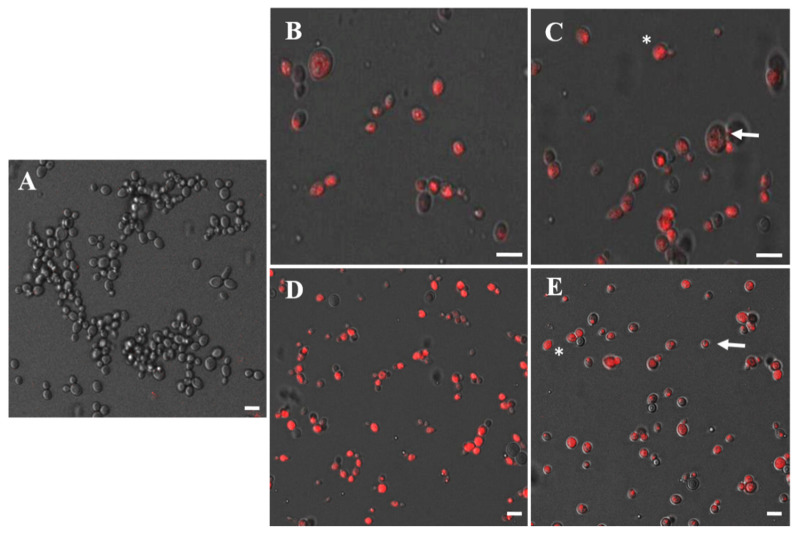
Confocal microscopy in *C. glabrata.* (**A**) Control *C. glabrata* CECT 1448. (**B**) 32 mg/L of the dendron 1, (**C**) combination 128 mg/L of the dendron 1 with 1 mg/L of amphotericin, (**D**) 64 mg/L of the dendron 2, (**E**) combination 128 mg/L of the dendron 2 with 4 mg/L of amphotericin. (Arrow: red dotted cells; asterisk: homogeneous red cells). Scale bar: 5 µm.

Cell death of *C. glabrata* CECT 1448 treated with dendrons was confirmed by confocal microscopy. In red, dead cells of *C. glabrata* are reflected. In Figure 4A the control hardly has any damaged cells, unlike the rest of the images. The MBDC concentrations of dendron 1 and dendron 2, 32 mg/L and 64 mg/L, respectively (Table 1), are visualized (Figure 4B,D). In both cases, the high degree of cell death (stained in red) and some living cells (unstained) were confirmed, the latter capable of growing on agar plates. This fact was not observed in the images obtained from the synergistic combinations of 128 mg/L of dendron 1 with amphotericin 1 mg/L (Figure 4C) and of 128 mg/L of dendron 2 with 4 mg/L amphotericin (Figure 4E), where 100% non-viable cells were verified.

The images allowed us to identify two different forms of propidium iodide (PI) internalization. Some cells appeared with several red dots inside (Figure 4C,E: arrow), while other cells had a completely red interior (Figure 4C,E: asterisk). Sangalli-Leite et al. [30] noted that cells treated with amphotericin could cause DNA degradation and that other studies have shown that it induces DNA condensation. PI cannot penetrate the cell membrane unless pores have been generated. Therefore, cells with dots could have a low permeability due to their higher resistance (persister cells) and require more time to increase the number of pores. These results could indicate that the treatment affects the DNA, which is fragmented and dispersed throughout the cytoplasm, visualized as red dots in the presence of PI. On the other hand, totally red cells could be those that offer less resistance to treatment. According to this statement/affirmation, it is logical to find a greater number of cells with red dots at concentrations where cell viability was completely reduced (0% viability), i.e., in combination treatments (synergistic and additive effect).

## 4. Conclusions

The dendritic compounds studied not only reduced or completely prevented the development of the biofilm but also inactivated and eradicated established biofilms. Therefore, they showed antifungal and antibiofilm activity against *C. glabrata.* The combination of these molecules with amphotericin gave excellent results both against biofilm development and established biofilms, managing to eliminate all cells at low concentrations from 2 to 10 times lower than concentrations required in individual treatments and reducing cytotoxicity. Consequently, these compounds could be a promising target of research to be used as *Candida* anti-biofilm agents on disinfectant solutions or to functionalize surfaces, such as catheters.

## Figures and Tables

**Table 1:** Anti-biofilm activity of dendrons and amphotericin tested against *C. glabrata* CECT1448.

	Biofilm Formation		Established Biofilm	
Molecules	MBIC (mg/L)	MFCB * (mg/L)	MBDC (mg/L)	MBEC * (mg/L)
Dendron 1	8	8–16	32	256–512
Dendron 2	8	8–16	64	512
Amphotericin	0.125	0.125	1	BNE (>128)

BNE: Biofilm not eradicated. * Drop plate method.

**Table 2:** FICI and percentage of viability of dendron 1 with amphotericin, and dendron 2 with amphotericin combinations against *C. glabrata* CECT1448. Results after 48 h treatment on preventing biofilm formation. Results analysis: Additive (A) and Synergy (S). (*): Use of Resazurin colorimetric assay.

**Dendron 1**					
Individual MBIC * **(mg/L)**		MBIC in Combination * **(mg/L)**			
**Dendron 1**	**Amphotericin**	**Dendron 1**	**Amphotericin**	**FICI**	Viability (%) ± SD *
8	0.125	1	0.06	0.61 (A)	0 ± 0
		4	0.06	0.98 (A)	0 ± 0
		0.5	0.06	-	17.8 ± 5.3
		0.25	0.06	-	19.4 ± 3.4
		4	0.03	-	19.7 ± 2.47
		2	0.03	-	21.7 ± 7.1
		1	0.03	-	35.3 ± 2.6
**Dendron 2**					
Individual MBIC * (mg/L)		MBIC in Combination * (mg/L)			
**Dendron 2**	**Amphotericin**	**Dendron 2**	**Amphotericin**	**FICI**	Viability (%) ± SD *
8	0.125	2	0.03	0.49 (S)	0 ± 0
		0.25	0.06	0.51 (A)	0 ± 0
		4	0.06	0.98 (A)	0 ± 0
		4	0.03	0.74 (A)	0 ± 0
		4	0.015	0.62 (A)	0 ± 0
		2	0.06	0.73 (A)	0 ± 0
		1	0.06	0.61 (A)	0 ± 0
		2	0.015	-	30.1 ± 2.5
		0.5	0.03	-	19.5 ± 3.4
		0.5	0.015	-	33.9 ± 5.8
		0.25	0.03	-	19.5 ± 2.7
